# Intasome architecture and chromatin density modulate retroviral integration into nucleosome

**DOI:** 10.1186/s12977-015-0145-9

**Published:** 2015-02-07

**Authors:** Mohamed Salah Benleulmi, Julien Matysiak, Daniel Rodrigo Henriquez, Cédric Vaillant, Paul Lesbats, Christina Calmels, Monica Naughtin, Oscar Leon, Anna Marie Skalka, Marc Ruff, Marc Lavigne, Marie-Line Andreola, Vincent Parissi

**Affiliations:** Fundamental Microbiology and Pathogenicity laboratory, UMR 5234, CNRS, University of Bordeaux, SFR transbiomed, Bordeaux, France; Virology program, ICBM, Faculty of Medicine, University of Chile, Santiago of Chile, Chile; Laboratoire de Physique, CNRS UMR 5672, Ecole Normale Supérieure, Lyon, France; Laboratoire Joliot-Curie, CNRS USR3010, Ecole Normale Supérieure, Lyon, France; Fox Chase Cancer Center, Philadelphia, USA; Département de Biologie Structurale intégrative, IGBMC (Institut de Génétique et de Biologie Moléculaire et Cellulaire), UDS, U596 INSERM, UMR7104, CNRS, Strasbourg, France; Institut Pasteur, Unité de virologie moléculaire et vaccinologie, UMR 3569, CNRS, Paris, France; Current address: Cancer Research UK, London Research Institute, Clare Hall Laboratories, Potters Bar, UK; Current address: Institut Cochin, INSERM U1016, CNRS UMR8104, Paris, France

**Keywords:** Retroviral integration selectivity, Intasomes, Nucleosomes, Chromatin

## Abstract

**Background:**

Retroviral integration depends on the interaction between intasomes, host chromatin and cellular targeting cofactors as LEDGF/p75 or BET proteins. Previous studies indicated that the retroviral integrase, by itself, may play a role in the local integration site selection within nucleosomal target DNA. We focused our study on this local association by analyzing the intrinsic properties of various retroviral intasomes to functionally accommodate different chromatin structures in the lack of other cofactors.

**Results:**

Using *in vitro* conditions allowing the efficient catalysis of full site integration without these cofactors, we show that distinct retroviral integrases are not equally affected by chromatin compactness. Indeed, while PFV and MLV integration reactions are favored into dense and stable nucleosomes, HIV-1 and ASV concerted integration reactions are preferred into poorly dense chromatin regions of our nucleosomal acceptor templates. Predicted nucleosome occupancy around integration sites identified in infected cells suggests the presence of a nucleosome at the MLV and HIV-1 integration sites surrounded by differently dense chromatin. Further analyses of the relationships between the *in vitro* integration site selectivity and the structure of the inserted DNA indicate that structural constraints within intasomes could account for their ability to accommodate nucleosomal DNA and could dictate their capability to bind nucleosomes functionally in these specific chromatin contexts.

**Conclusions:**

Thus, both intasome architecture and compactness of the chromatin surrounding the targeted nucleosome appear important determinants of the retroviral integration site selectivity. This supports a mechanism involving a global targeting of the intasomes toward suitable chromatin regions followed by a local integration site selection modulated by the intrinsic structural constraints of the intasomes governing the target DNA bending and dictating their sensitivity toward suitable specific nucleosomal structures and density.

**Electronic supplementary material:**

The online version of this article (doi:10.1186/s12977-015-0145-9) contains supplementary material, which is available to authorized users.

## Background

The retroviral replication requires the integration of viral DNA into the host genome. This step, catalyzed by retroviral integrase (IN), is performed within a nucleoprotein structure, the pre-integration complex (PIC), which must reach the nucleus and gain access to the target DNA in a nucleosomal compacted structure [1,2]. During the integration process, IN first removes two nucleotides from both 3′ ends of the long terminal repeats generated during reverse transcription, which is called the 3′ processing reaction. The newly exposed 3′OH groups are then used in the concerted nucleophilic attack of two phosphodiester bonds across the major groove of the host DNA, a reaction termed joining or strand transfer [3-6]. Post-integration DNA reparation (PIR) of this integration intermediate, leading to the establishment of the provirus, generates short target DNA duplications. The size of these duplications varies among retroviruses: 4 bp in the case of the *Spumaretrovirus*, prototype foamy virus (PFV) and the *Gammaretrovirus*, murine leukemia virus (MLV), 5 bp for the *Lentivirus,* human immunodeficience virus (HIV) and 6 bp for the *Alpharetrovirus,* avian sarcoma virus (ASV) and some *Beta-* and *Deltaretroviruses* [7]. This size corresponds to the distance between the phosphodiester bonds of cellular DNA attacked by the two viral DNA ends during concerted integration process. This distance is dictated by physical constraints within the intasomes, as the space between the two functional catalytic sites, governing the bending of the target DNA [8-10].

Retroviral INs comprise three distinct structural and functional domains: the N–terminal domain (which is preceded by an additional domain, the N-terminal extension domain (NED), in Spumaretroviral, Gamma- and Epsilonretroviral INs), adopts an HTH-fold and is characterized by the presence of an HHCC zinc finger–like motif; the core domain, structurally related to *Escherichia coli* RNase H and other polynucleotidyl-transferases, contains the invariant acidic triad DDE involved in the coordination of the catalytic cofactors; and the C–terminal domain, the least conserved among retroviral INs, includes an SH3-fold [11,12] for recent reviews). Although numerous partial structures of INs from different retroviral genera have been determined, only PFV IN has been crystallized in its full-length form, in the presence of its DNA substrates, providing unprecedented details on the organization of the successive nucleoprotein complexes involved in the integration process, from the stable synaptic complex (SSC, also referred to as the intasome) to the strand transfer complex (STC) [8-10]. In agreement with these structural data, previous biochemical studies performed on INs from different retroviruses have also concluded that the integration reaction was carried out by an IN tetramer [13-15], although the global architecture of the intasome and the STC might vary from a system to another [16,17]. Although IN alone can catalyze integration *in vitro,* other cellular or viral proteins have been found to play an important role in infected cells within the pre-integration complex (PIC) or during transit to the nucleus (for a review on the IN cofactors see [18]). Furthermore some post-translational modifications of HIV-1 IN have been reported that could also affect the enzyme activity and its cellular behavior [19,20].

The integration boundaries mark the definitive position of the provirus, and the site selection is highly important for the outcome of the infection. Integration into a region of active transcription promotes viral gene expression, whereas integration into transcriptionally repressed chromatin could potentially promote viral latency [21-23]. If HIV-1 DNA is integrated into actively transcribed genes, the viral genes would need to be repressed to allow persistence of the infection. The mechanisms that control viral gene expression are not yet understood fully. Several factors are involved in the infection of latent and resting cells and in the preferential integration found at the periphery of the nucleus in such latent T cells [24-26]. On the other hand, for the host, integration events can lead to the activation of proto-oncogenes or the inactivation of essential cellular genes [27]. For all these reasons it appears essential to elucidate the molecular mechanisms governing integration site selection to set up safe and efficient gene therapy approaches.

To this end, genome-wide studies have been undertaken and have revealed that retroviruses target different regions of the host chromatin. These studies have shown that some members of the *Spumaretrovirus* and *Gammaretrovirus* families favor transcription start sites of actively transcribed genes (TSSs) and CpG islands, the *Lentiviruses* that have been studied integrate preferentially into active transcription units (TUs), the *Alpha*– and *Deltaretroviruses* tested exhibit only a weak preference for TUs, and some *Betaretroviruses* integrate in an almost random fashion (reviewed in [28]). Globally, retroviruses can thus be classified into three separate categories: those that preferentially target TSSs and CpG islands, those that display a strong bias toward integration into TUs and those that exhibit little or no particular preference for any chromosomal feature. Interestingly these three groups comprise viruses that share the same target site duplication signature as mentioned above. This raises the question of the relationship between these genomic signatures, closely linked to intasome structures and target DNA bending, and integration selectivity.

HIV-1 and MLV integration site preferences have been shown to rely essentially on the interaction of IN with specific host cell factors - *i.e*. lens epithelium-derived growth factor (LEDGF/p75) (see [29] for a review) and bromodomain and extraterminal domain (BET) proteins [30,31], respectively - that recognize defined transcription-associated histone modifications. On the other hand, cellular data obtained from a murine LEDGF/p75 knock-out (KO) model have shown that LEDGF/p75 was not involved in the local target DNA sequence preference associated with HIV-1 integration [32-34]. Thus, despite its ability to bind histone tails through its PWWP domain [35], LEDGF/p75 is unlikely the sole protein responsible for targeting integration into nucleosomal DNA which may require additional intasome/nucleosome interactions and/or chromatin-remodeling activities [36]. Recently it has been shown that dissociating the interaction between MLV integrase and BET proteins did not change the local integration site sequence selection [37]. Furthermore, in the lack of LEDGF/p75, the local HIV-1 integration sequence selectivity was also shown unchanged [38,39]. Taken together all these data suggest that integrase protein plays a role in the local targeting to the nucleosomal locus after targeting of the intasome into suitable regions of the chromatin thanks to its interaction with cellular cofactors. We, thus, focused our work on the better determination of this final local association between IN and nucleosomes.

The functional interaction between retroviral intasomes and nucleosomes in cells remains elusive. There are no preferential positions for nucleosomes across the majority of the human genome [40]. Consequently, the dynamic nature of chromatin makes it difficult to accurately predict the positions of nucleosomes in the cell. For this reason, *in vitro* approaches based on the use of chromatinized templates, in which nucleosome positioning is exclusively sequence-driven, are valuable for understanding the specific influence of the chromatin structure on retroviral integration site selectivity. Early results showed that DNA distorsion and nucleosome could favor retroviral integration *in vitro* [41-46]. More recently, *in vitro* study using chromatinized template has shown that HIV-1 integration into nucleosomes could be modulated by chromatin structure and dynamics [36]. Additionally, partial one end and physiological two ends concerted HIV-1 integration reactions were shown to be affected differently by chromatin *in vitro* suggesting that the structure of the different intasomes catalyzing these various integration reactions could impact their sensitivity to nucleosomal DNA [36].

In order to determine how integration could be modulated both by chromatin structure and intasome architecture, we compared the ability of various retroviral intasomes to accommodate different chromatin structures both *in vitro* and *in vivo.* We selected retroviral integrases that vary in their integration site preference in infected cells and in the size of the duplicated target site sequence governed by the distance separating the two active sites of the intasome and, thus, the architecture of the IN catalytic pocket. To determine the intrinsic sensitivity of the selected integrases toward chromatin compactness was determined by analyzing their integration efficiency, selectivity and fidelity in naked and nucleosomal DNA *in vitro* using specific biochemical conditions allowing efficient full site integration reactions in the lack of cofactors. Our results indicate that the impact of the chromatin structure at the integration site is mainly driven by intrinsic physical constraints within the retroviral intasomes. These constraints could thus dictate their capability to bind nucleosomes functionally in specific chromatin contexts after targeting into specific regions of the host genome via the interaction with cellular cofactors.

## Results

### *In vitro* integration catalyzed on chromatin by integrases from various retroviral genera

**Figure 1:**
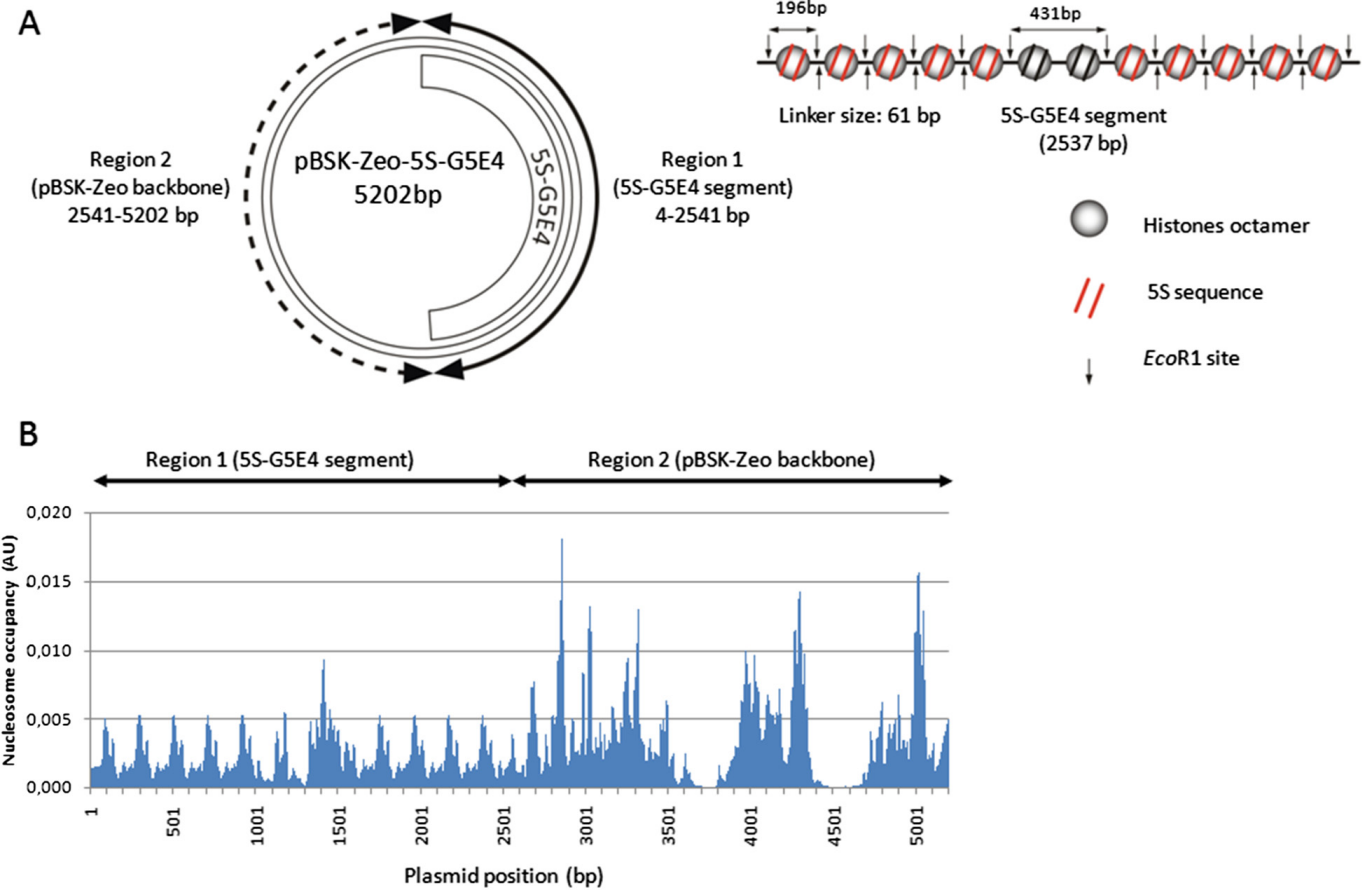
**pBSK-Zeo-5S-G5E4 (p5S) acceptor plasmids used in the work.** The 2.56 kb 5S-G5E4 fragment DNA for polynucleosome assembly (PN) was previously described [62] and was cloned into the pBSP-zeo vector **(A)**. This fragment is made of two times five repeats of 5S sequences surrounding a central sequence containing five gal4 DNA binding sites and the adenovirus 2 E4 minimal promoter. The p5S vector thus contains a region 1 containing nucleosome-positioning sequences and a region 2 containing the pBSK-zeo backbone. Each 5S fragment is separated by two *Eco*RI restriction sites. Nucleosome occupancy prediction performed using the method previously described by [53] and used in [36] indicates the formation on the chromatinized vector of two regions with different chromatin organization (regular and stable nucleosomes in the 5S-G5E4 region 1 and less organized and stable nucleosomes in the pBSK-Zeo backbone region 2 **(B)**. Restriction inhibition assay demonstrates that the two regions are not equally accessible since region 1 is highly protected when chromatinized whereas region 2 remains highly accessible for restriction site cutting even in the nucleosomal structure (see Additional file 1: Figure S1).

HIV-1, PFV, MLV and ASV INs were compared using their specific donor DNAs (described in Additional file 1: Figure S1) and p5S acceptor plasmids derived from the previously described pBSK-Zeo-S5G5E4 receptor vector (Figure 1A and Additional file 2: Figure S2A) containing a 5S-G5E4 fragment carrying two times five repeats of 5S sequences surrounding a central sequence containing five gal4 DNA binding sites and the adenovirus 2 E4 minimal promoter. This construct allows the *in vitro* association of nucleosomes in stable, regularly spaced and defined positions of the DNA template leading to dense polynucleosome (PN) [36]. A nucleosome positioning prediction algorithm performed on the acceptor plasmid sequence (Figure 1B) shows that it contains two regions allowing the formation of different chromatin structure after nucleosomes assembly. Nucleosomes assembly in region 1, containing the 5S-G5E4 fragment, leads to dense, highly organized and stable chromatin. In contrast, assembly in region 2, corresponding to the pBSK-Zeo backbone, allows the formation of nucleosomes in a less dense, more dynamic and less organized chromatin structure.

The overall chromatin structure of the plasmid was first checked by DNase 1 protection after nucleosomes assembly with increasing amounts of the native core histones, H3, H4, H2A and H2B (Additional file 2: Figure S2B). Then, typical restriction assays (REA) using restriction enzymes cutting distinctively in both regions (see Additional file 2: Figure S2A and S2C) showed that the 5S-G5E4 containing domain 1 was poorly accessible, unlike region 2 which remained highly sensitive to DNA cleavage. This confirmed the two distinct chromatin structural regions (regions 1, densely occupied and region 2 sparsely occupied), as predicted from the nucleosome positioning prediction algorithm. Finally, the accurate nucleosome positioning in the 5S-G5E4 region 1 was confirmed by agarose nucleosome gel shift assay performed on *Eco*RI cleaved 5S fragments (Additional file 2: Figure S2D), demonstrating the formation of evenly spaced mononucleosomes of the same size accurately positioned in known sites.

**Figure 2:**
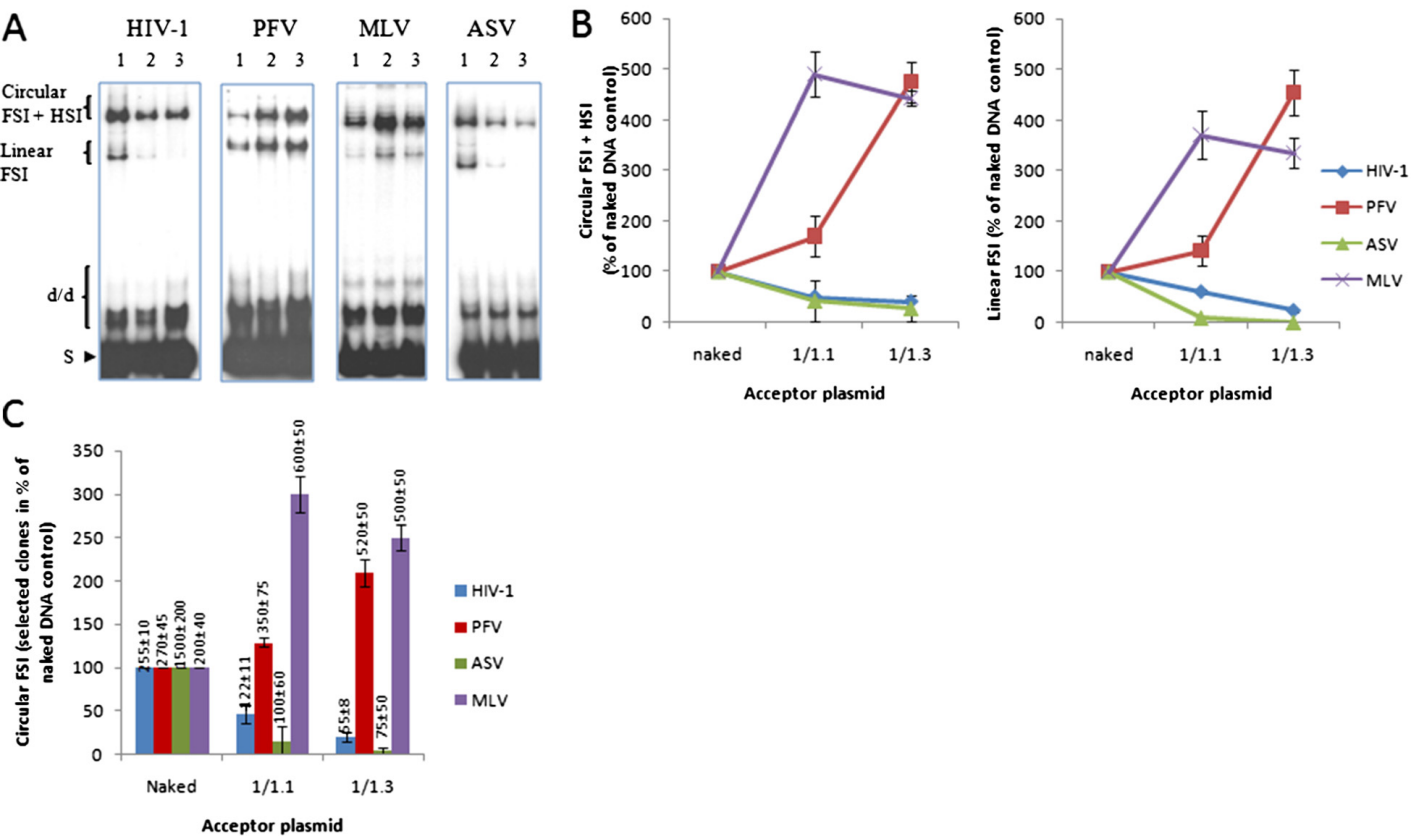
**HIV-1, PFV, MLV and ASV** ***in vitro*** **integration on naked and chromatinized p5S vectors.** Concerted integration assay was performed with 10 ng of donor DNA and 100 ng of p5S naked plasmids (lanes 1), or polynucleosomal vectors assembled with increasing amounts of histones expressed as DNA/histones mass ratio (μg/μg) (1/1.1, lanes 2; 1/1.3, lanes 3) and either HIV-1, PFV, MLV (100nM) or ASV (600 nM) integrases. The reaction products were loaded onto 1% agarose gel and a representative set of experiments is shown in the figure **(A)**. The position and structures of the donor substrate and different products obtained after half-site (HSI), full-site (FSI) and donor/donor integration (d/d) are shown. The circular FSI + HSI and linear FSI products were quantified on gel using the ImageJ software and are shown respectively in the left and right panels in **(B)**. The circular FSI products were specifically quantified by cloning in bacteria and shown as the number of ampicillin-, kanamycin- and tetracycline-resistant selected clones as a percentage of integration reaction control performed with naked vectors **(C)**. All the values are shown as the mean ± standard deviation (error bars) of at least three independent sets of experiments. The number of selected clones is also shown at the top of the histogram.

In order to compare the different retroviral INs enzymes were all purified following similar purification procedure derived from previously published work [47] and tested under similar reaction conditions when possible. We first selected reaction conditions allowing efficient concerted integration for all the enzymes tested in the work. Two distinct assays have been reported by different groups to reproduce efficiently concerted integration *in vitro.* The first assay uses long viral DNA substrates and PEG and allows the detection of concerted integration in the absence of LEDGF/p75 but show poor stimulation effect of the cofactor [48,49]. The second assay developed by Cherepanov group does not use PEG and allows the detection of LEDGF/p75 dependent stimulation of concerted integration but poor full site integration in the absence of the cofactor [50,51]. Consequently, since our main aim was to analyze integration in the lack of cofactors, we have chosen to use long donor substrates in presence of PEG. These conditions allowed us to test HIV-1, PFV and MLV INs using the same final protein concentration of 100 nM. Blunt-end donor DNAs containing the specific viral ends U3 and U5 were used in concerted integration assays performed using naked or chromatinized p5S acceptor. As reported in Figure 2A and B, under these conditions all the enzymes were found highly active using their specific donor DNA and the naked p5S receptor plasmid. Specific isolation and quantification of the physiological FSI integration products (Figure 2C) show that the enzymes were equally active leading to a similar amount of integrants (200 to 225 per experiment). However, despite their similar activity on naked DNA, large differences were found in their activity on nucleosomal templates. Indeed, in contrast to HIV-1, the activity of PFV and MLV INs were strongly stimulated on chromatinized receptor plasmid leading to a 4- to 5-fold increase in the integration products (Figure 2A as well as quantification in 2B and C). No significant change in integration fidelity was found using the chromatinized vector comparing to the naked one (Additional file 3: Figure S3).

To confirm that different retroviral INs could be affected differently by chromatin *in vitro*¸ ASV enzyme was tested under its previously reported optimal conditions [52]. Under these conditions, ASV IN was found to be more active on naked DNA than HIV-1, PFV and MLV enzymes catalyzing the formation of a greater amount of integration products (Figure 2A and quantification in 2B). Cloning and quantification of the circular FSI forms, the most representative for the physiological integration reaction observed in cells, confirmed that ASV IN was more active *in vitro* (Figure 2C) and preferentially displayed the expected 6 bp target site duplication (Additional file 3: Figure S3). However, despite its higher activity found on naked DNA, ASV integration was strongly inhibited by nucleosomes assembly as observed for HIV-1 IN (see Figure 2A and quantification in 2B). Additionally, specific quantification of the circular FSI products also revealed a strong decrease in the number of integrants on nucleosomal templates even at low histones concentrations (Figure 2C). Similar results were obtained using different enzyme constructs, preparations, purification procedure and various viral DNA substrates (see data reported for HIV-1 IN in Additional file 4: Figure S4).

To better ascertain the relationship between integration, nucleosomes positions and chromatin structure, we mapped more precisely the integration sites obtained from retroviral integrases on the different chromatin structures of the acceptor plasmid.

### Effect of nucleosomes density on *in vitro* HIV-1, PFV, ASV and MLV integration selectivity

**Figure 3:**
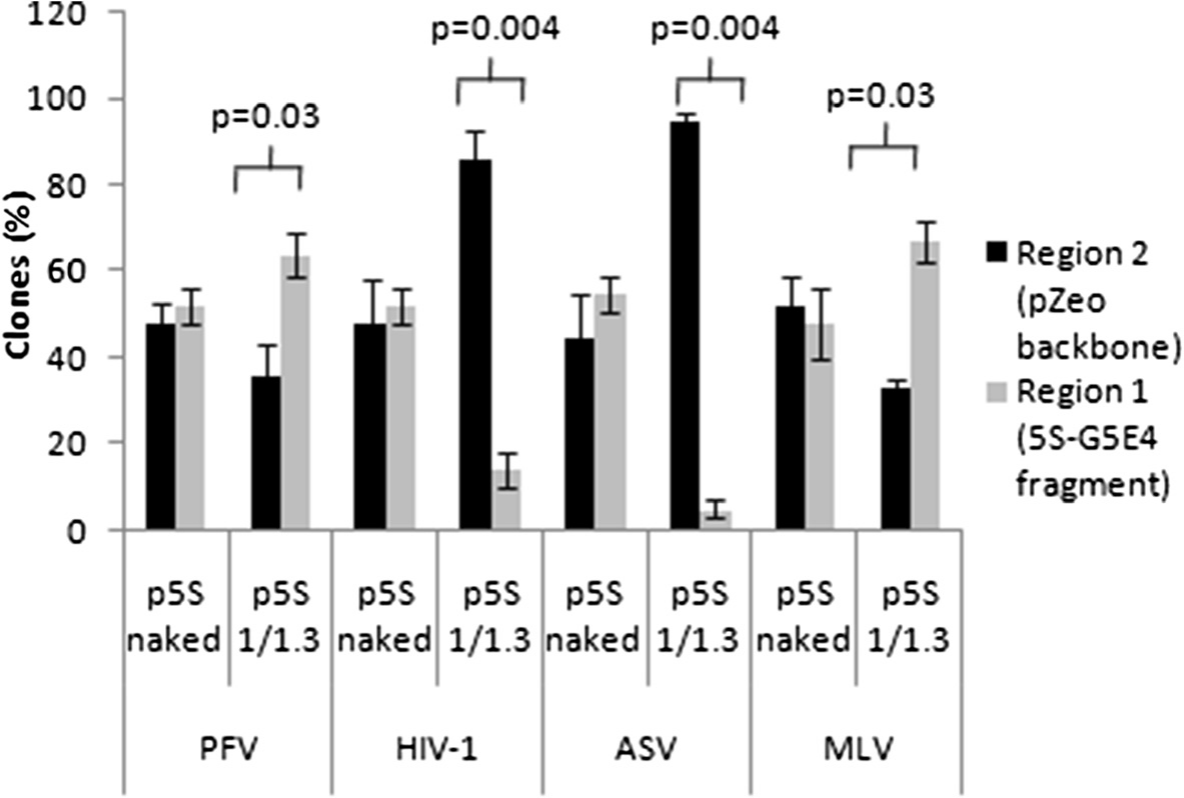
**Localization of the HIV-1, PFV, MLV and ASV integration sites in both regions of the naked or chromatinized acceptor DNA.** Fifty PFV, HIV-1, ASV and MLV integrants carrying the correct target DNA duplications (respectively 4, 5, 6 and 4 bp) were selected from the concerted integration shown in Figure 2 using p5S vector, either under the naked structure or chromatinized with a DNA/histones ratio of 1/1.3. The positions of the different integration events were identified and shown in region 1 or region 2. The values are plotted as the number of ampicillin-, kanamycin- and tetracycline-resistant selected clones as a percentage of integration reaction control performed with naked vectors and as the mean ± standard deviation (error bars) of at least three independent sets of experiments. The number of selected clones is also shown at the top of the histogram. A Student test was performed on serial values and the significant p values are reported in the figure.

The integration site targeting in the two regions of the plasmid differing in their nucleosomes density and stability was first studied. Fifty integrants previously obtained with naked and chromatinized p5S acceptors using HIV-1, PFV, ASV and MLV IN were cloned, sequenced and the positions of the integration sites were compared. As shown in Figure 3, most of the HIV-1 integration sites mapped on the chromatinized templates were found in the region of low nucleosome occupancy outside the 5S-G5E4 nucleosome positioning region 1 (86% of total analyzed sites), thus confirming previously reported results [36]. Similar profile was observed with ASV integrase with a more drastic redistribution of the integration sites in the lower nucleosome density region of the chromatinized vector. However, in contrast to HIV-1 and ASV integration, the 5S-G5E4 positioning region 1 of the plasmid containing dense nucleosomes was not found refractory to PFV and MLV integration. Indeed these INs were found to accommodate both regions of the plasmid with a significant preference for the nucleosome dense region 1 (64% of sites analyzed were found within the 5S-G5E4 fragment for PFV and 74% for MLV).

**Figure 4:**
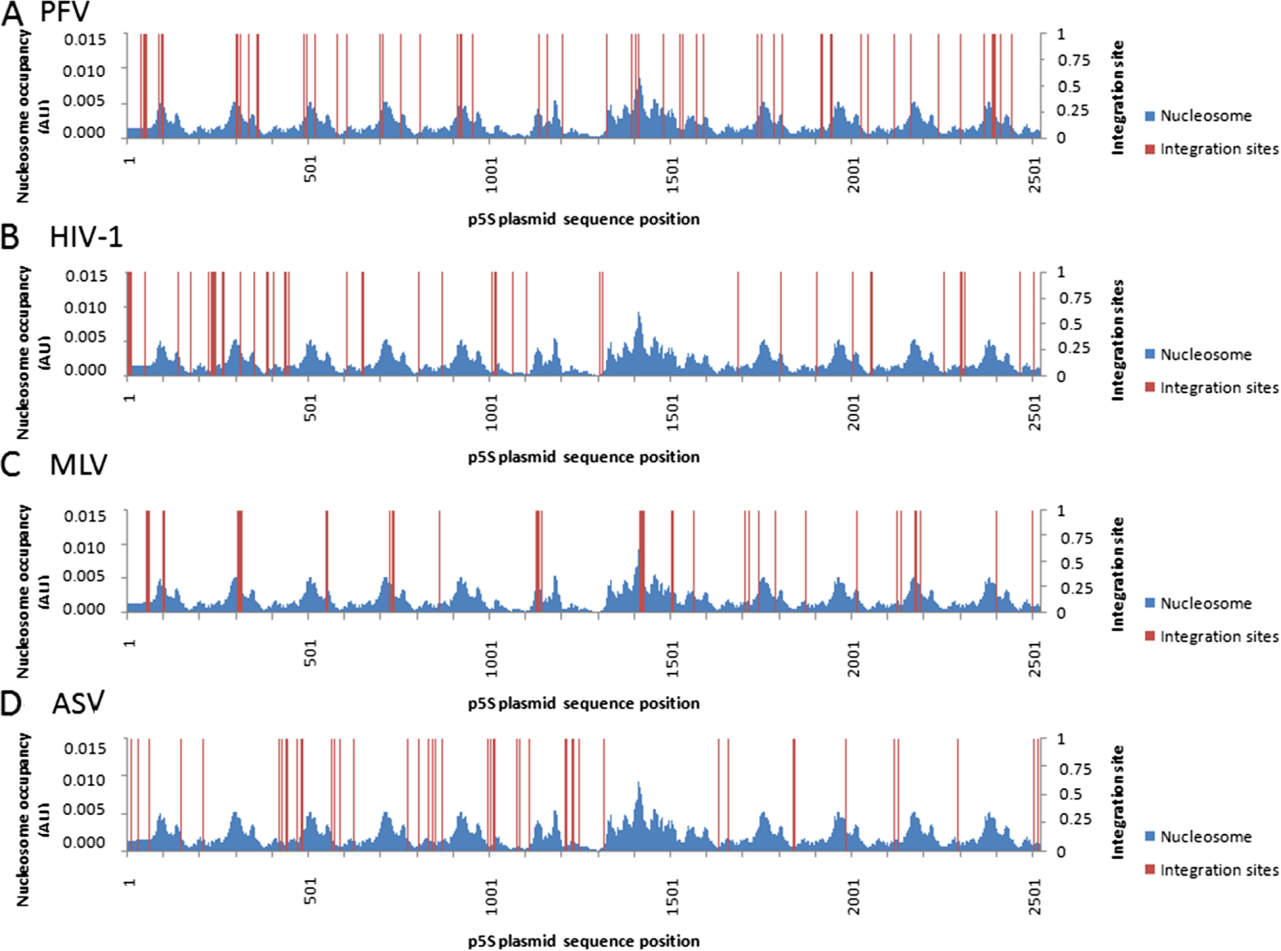
**Comparison of HIV-1, PFV, MLV and ASV integration sites and nucleosome occupanc** ***in vitro*** **.** Fifty PFV **(A)**, HIV-1 **(B)**, MLV **(C)** and ASV **(D)** integrants carrying the correct target DNA duplication obtained after integration assay carried on the p5S vector chromatinized with a 1/1.3 DNA/histones ratio and localized in the 5S-G5E4 fragment were positioned onto the DNA sequence and compared to the nucleosome occupancy determined using the method previously described by [53] and used in [36].

**Figure 5:**
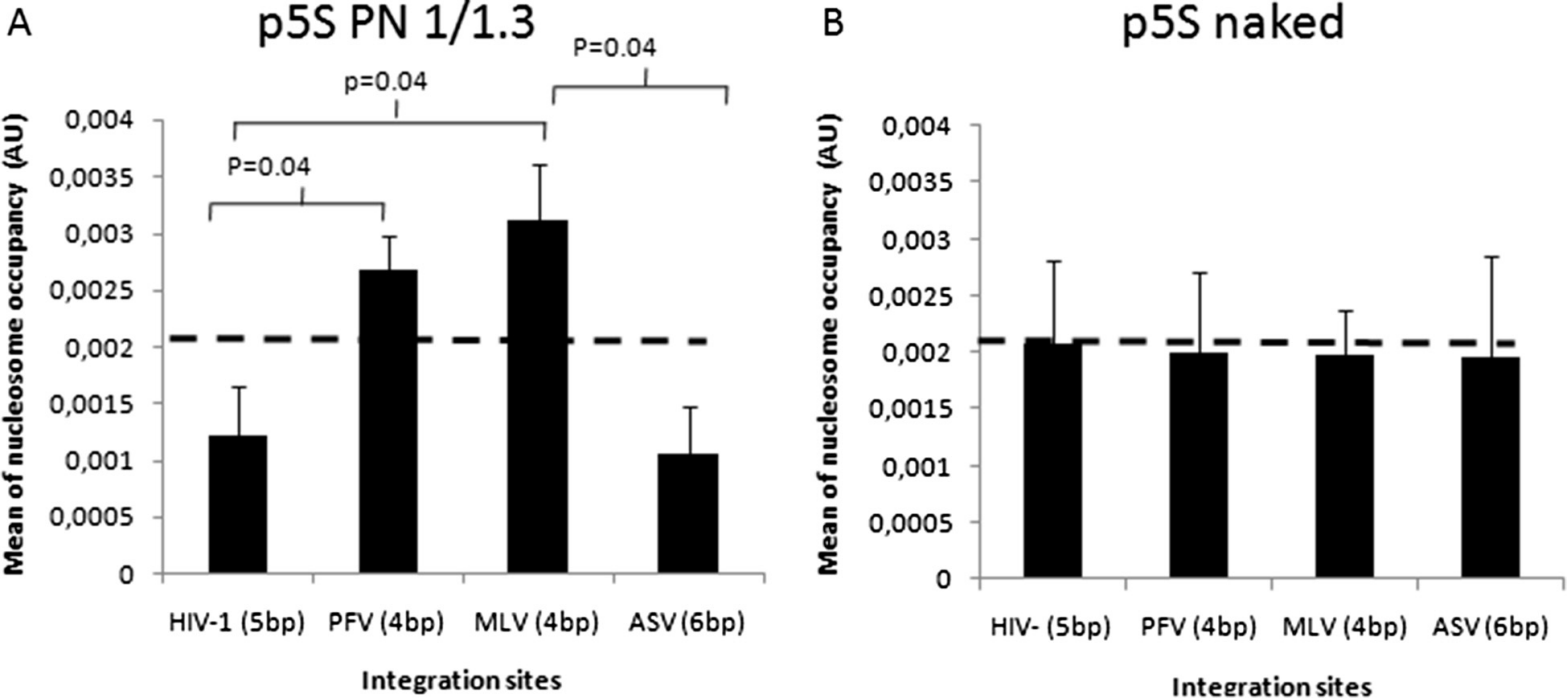
**Correlation between HIV-1, PFV, MLV and ASV integration sites and nucleosome occupancy means** ***in vitro*** **.** The mean nucleosome occupancy found at the integration site on chromatinized or naked plasmid was calculated and plotted respectively in **(A)** and **(B)**. The mean nucleosome occupancy of the overall plasmid is reported as dotted line. A Student test was performed on serial values and the significant p values are reported in the figure.

To correlate more precisely the nucleosomes positions with integration sites we analyzed more in details their localization in the 5S-G5E4 region 1 containing nucleosomes assembled at known positions predicted from the algorithm described in [53] (and derived from [54]) and experimentally validated (see Additional file 2: Figure S2). The analysis was performed on 50 integration sites previously selected within the 5S-G5E4 region for each enzyme. PFV and MLV sites were found to be preferentially enriched in regions of high probability of nucleosome presence (see Figure 4A and 4C), whereas no bias in the integration site positions was found on the naked version of the plasmid for both enzymes (Additional file 5: Figure S5). The calculation of the mean nucleosome occupancy found at the integration sites confirms a strong preference of MLV and PFV integration for nucleosomes in the dense region of the reconstituted chromatin template (Figure 5A). Sequence bias was ruled out since the integration in the naked plasmid did not show such specific localization in regions favoring nucleosomes positioning (Figure 5B). Consequently, our data show that MLV and PFV *in vitro* integration reactions are favored on nucleosomes localized in dense and stable chromatin region.

HIV-1 and ASV integration sites carrying the physiological duplications (respectively 5 and 6 bp) obtained under the same reaction conditions and using the p5S vector assembled with a similar 1/1.3 DNA/histones ratio (w/w) were then analyzed. The integration experiment was reproduced a sufficient number of times to obtain a similar amount of 50 integrants carrying integration sites in the 5S-G5E4 region for both enzymes. As shown in Figure 4B and D, a strong bias toward regions of low nucleosome occupancy, especially the linker regions separating the nucleosomes, was found in the 5S-G5E4 region. The mean nucleosome occupancy found at the HIV-1 and ASV integration sites was 2-fold lower than the global mean found for the overall plasmid sequence (Figure 5A). Again, this distribution was not due to a sequence bias since a random distribution was found in the naked plasmid (Additional file 5: Figure S5 and Figure 5B). This indicates that both HIV-1 and ASV integration are preferred in regions containing poorly dense nucleosomes and that nucleosomes localized in organized and stable regions are not favored substrates for HIV-1 and ASV *in vitro* integration in contrast to MLV and PFV.

As shown in Additional file 3: Figure S3 and Additional file 6: Figure S6A HIV-1 IN also catalyzed *in vitro* integration events displaying 4 bp target site duplications, in addition to the correct 5 bp duplications. As discussed below and shown in [14], these integration events are assumed to be catalyzed by differently structured intasomes. While no bias was found in the distribution of the 4 bp integration loci in the naked DNA (Additional file 6: Figure S6B), a strong bias for the 5S region was observed for these events (Additional file 6: Figure S6C) with a strong preference for nucleosomes dense regions of the plasmid. Since these 4 bp and 5 bp integration events were catalyzed by intasomes from the same enzyme preparation this result strongly suggests that the differences found in the sensitivity of the various INs for nucleosomes are not due to differences in reaction conditions or activity. Moreover these data also suggest that the differences found in nucleosome sensitivity between the various integrases are mainly due to local constraints in the catalytic pocket of the various intasomes that define the target DNA duplication.

### *In vitro* PFV IN sensitivity to nucleosome is not dependent on its N-terminal extension domain (NED)

Since MLV and PFV integrases both contain an additional N-terminal extension domain (NED) compared to HIV-1 IN and ASV [8], we wondered whether it could be responsible for the difference in the impact of chromatin structures on *in vitro* integration. A PFV IN deleted for this NED domain was thus expressed and purified according to the same procedure as for wt IN and its *in vitro* integration activity was compared using naked and chromatinized templates. Wt and the NED-deleted INs were found to be equally active under standard conditions using the naked p5S acceptor plasmid, as it was expected from previous data obtained using short donor DNA [47]. This indicates that both enzymes share a similar functional structure even on long donor DNA. Analysis of the integration site structure also indicated that both enzymes shared the same target site duplication of 4 bp (Additional file 3: Figure S3), confirming that this duplication is mainly governed by the arrangement of viral ends in the catalytic sites involved in the strand transfer reaction.

**Figure 6:**
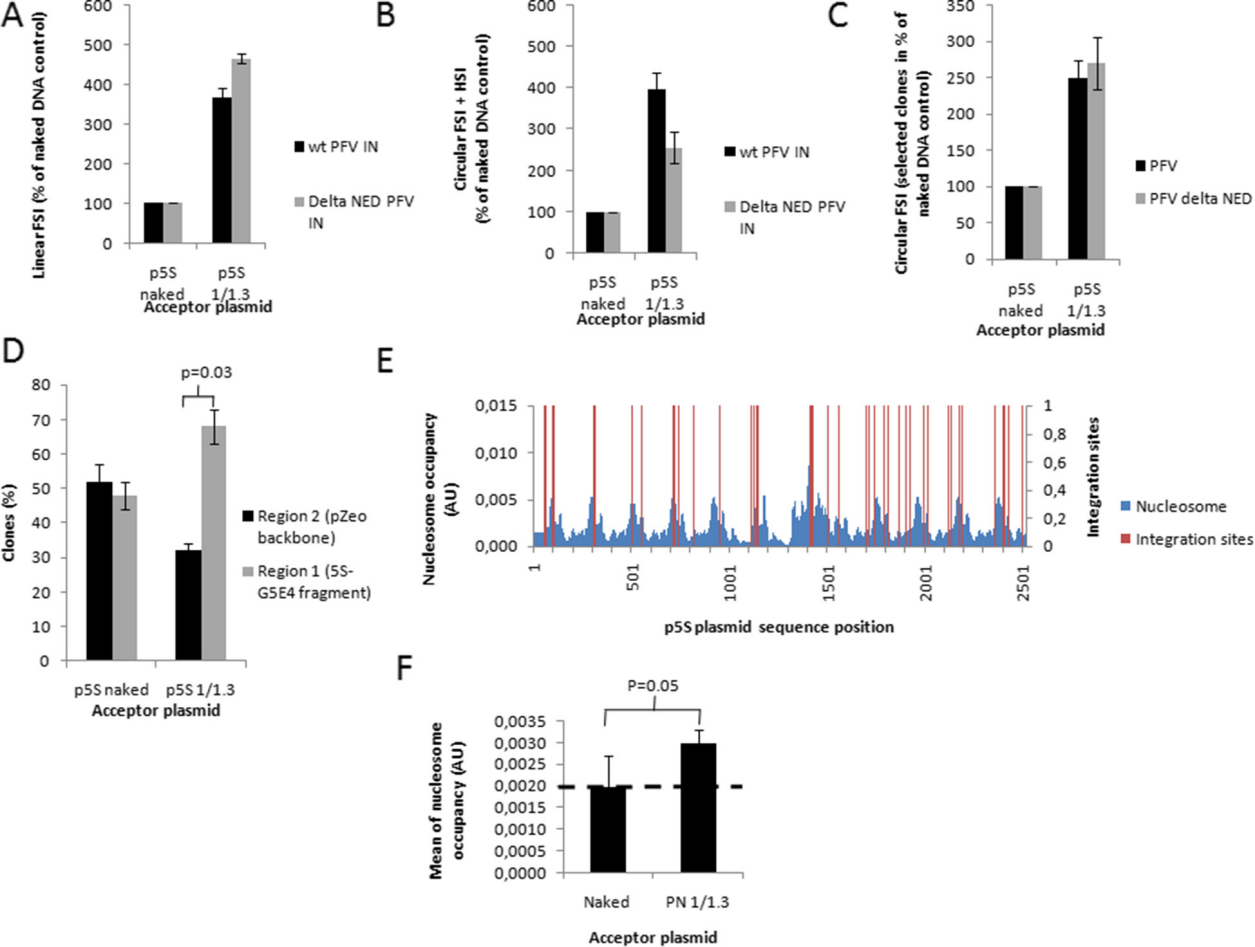
***In vitro*** **integration catalyzed by WT and delta NED PFV INs on naked and chromatinized p5S vectors.** Concerted integration assay was performed with 10 ng of donor DNA and 100 ng of p5S naked plasmids (lanes 1), or p5S polynucleosomalp5S vectors assembled with a 1/1.1 DNA/histones ratio (μg/μg) either 90nM wt PFV IN or 90nM delta NED PFV IN. The reaction products were loaded on 1% agarose gel and the linear FSI and circular FSI + HSI products were quantified on gel autoradiography using the ImageJsoftware and the values are plotted respectively in **(A)** and **(B)**. The circular FSI products were specifically quantified by cloning in bacteria and plotted as the number of ampicillin-, kanamycin- and tetracycline-resistant selected clones in percentage of integration reaction control performed with naked vectors **(C)**. The positions of the different integration events were then identified by sequencing and plotted in region 1 or region 2 **(D)**. Values correspond to the mean ± standard deviation (error bars) of 3 independent sets of experiments. The number of selected clones is also shown at the top of the histograms. Fifty integrants carrying the correct 4 bp target DNA duplication obtained after integration assay carried on the p5S vector chromatinized with a 1/1.3 DNA/histones ratio were localized on the p5S sequence **(E)** and compared to the nucleosome occupancy determined using the method previously described by [53] and used in [36]. The mean nucleosome occupancy found at the integration site on chromatinized or naked plasmid was calculated and plotted respectively in **(F)**. A Student test was performed on serial values: *p < 0.05.

When the two enzymes were assayed on a chromatinized p5S acceptor template under optimal stimulation with regards to integration into naked vector, the same stimulation profile was observed. Indeed, nucleosomes induced a strong stimulation of all the hetero-reaction products, leading to a 3- to 4-fold increase in both linear FSI (Figure 6A) and circular FSI + HSI (Figure 6B) products. Cloning and quantification of the physiological circular FSI integration products confirmed that both enzymes were stimulated by nucleosomes assembled on the target templates (Figure 6C). The position of the integration sites in the two regions of the p5S plasmid showed a similar distribution between both enzymes with a broad localization throughout the plasmid and a significant preference for the 5S region (compare Figure 6D and Figure 3). We next analyzed the PFV integration site localization in the nucleosome-enriched 5S-G5E4 region of the plasmid and compared it to the predicted nucleosome position. As shown in Figure 6E, the distribution of the delta NED enzyme was similar to that of the wild type integrase (compare Figure 4A and Figure 6E), whereas no relevant sequence biases were detected in the naked vector (see nucleosome occupancy means in Figure 6F). Taken together, our data demonstrate that the NED domain from PFV integrase was responsible neither for the nucleosomes mediated stimulation of *in vitro* integration catalyzed by this enzyme, nor for its *in vitro* selectivity toward nucleosomal DNA.

### Comparison between integration sites in infected cells and predicted nucleosome occupancy

In order to investigate whether the sensitivity to dense nucleosomes regions found *in vitro* for the retroviral enzymes could be relevant in a more physiological context we analyzed the positions of cellular integration sites from two retroviruses showing distinct and opposite *in vitro* selectivity. HIV-1 and MLV were chosen since they present highly divergent *in vitro* and *in vivo* integration profiles. Furthermore their integration profiles in infected cells were extensively reported and, thus, a sufficient number of integration sites could be recovered from literature allowing a statistically relevant analysis.

The sequence surrounding 41 435 and 32 631 non redundant HIV-1 and MLV integration sites respectively obtained in human infected cells and selected from previous studies [55,56] were mapped into the human genome and submitted to a nucleosome-position prediction algorithm developed previously by Milani *et al. *[53]. This algorithm was chosen since it has been shown to provide predictions that are in good agreement with nucleosome positioning determined *in vitro* by physical and biochemical approaches [53,57,58]. This has been further validated on our *in vitro* receptor templates by showing a good correlation with the experimentally determined nucleosomes positions (see Additional file 2: Figure S2). Actually, the performance of this method is as good as the more commonly used approach developed by Segal and Widom’s groups [59] (the mean Pearson correlation between genome-wide experiments and prediction is > 0.75).

**Figure 7:**
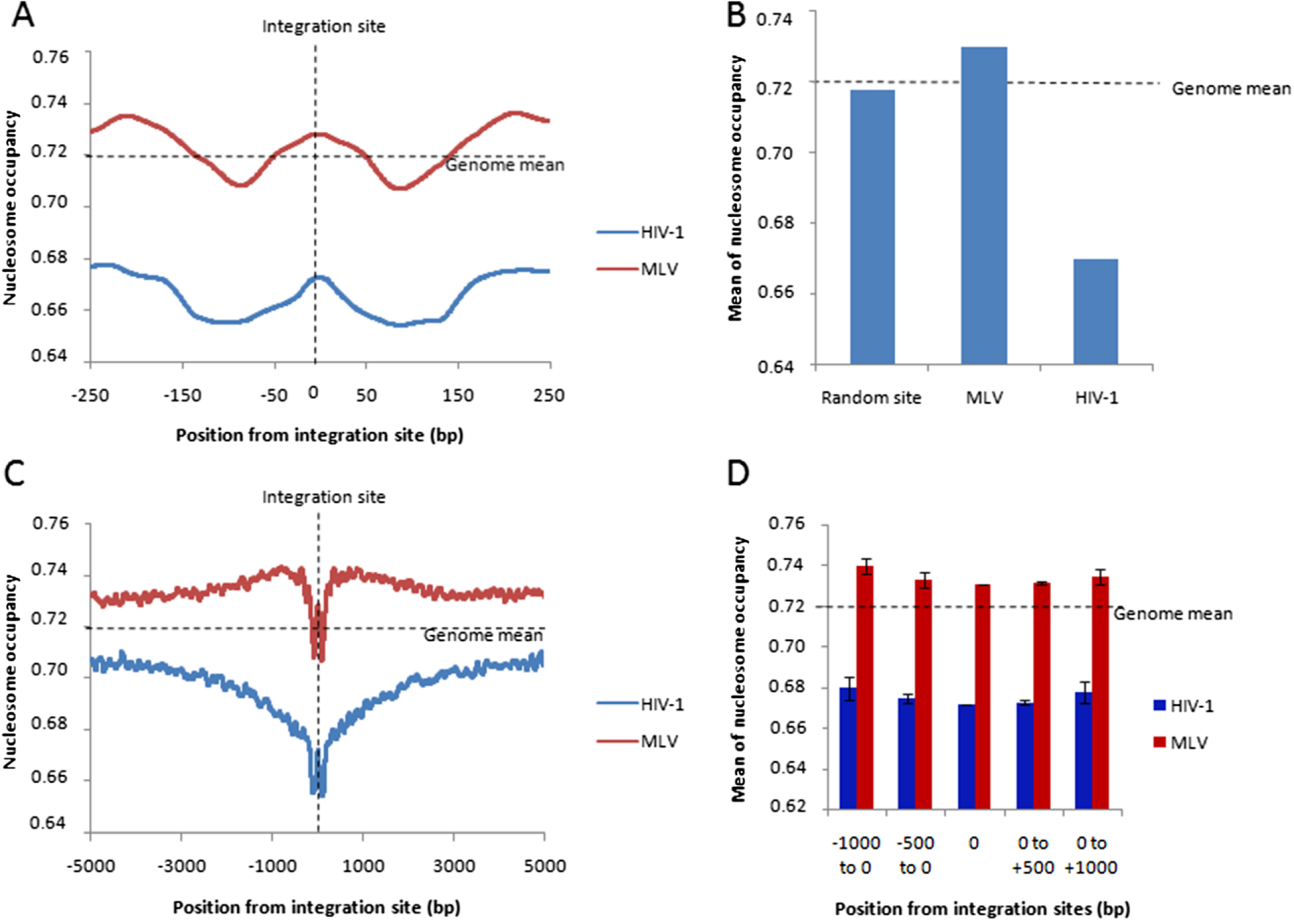
**Correlation between** ***in vivo*** **HIV-1 and MLV integration sites positions and nucleosome occupancy.** 41 435 HIV-1 integration loci obtained from [12], and 32 631 MLV integration sites obtained from [56] were plotted to human genome and submitted to the nucleosome-positioning prediction analysis set up previously [39]. The means of nucleosome occupancy surrounding the integration site (between −250 bp and 250 bp) are reported in **(A)**. The means of nucleosome occupancy at the integration site are reported in **(B)** for MLV, HIV-1 and randomly generated insertion sites. The means of nucleosome occupancy in the region between −5 000 bp to + 5 000 bp from the integration site are reported in **(C)**. Results are the means of the analyses performed from the 41435 HIV-1 and 32 361 MLV integrants. The means of nucleosomes occupancy in −1000 to 0, −500 to 0, 0 to 500 and 0 to 1000 bp regions surrounding the integration sites are reported in **(D)**. Dotted lines represent the position of the integration sites and the global occupancy mean calculated for the entire genome.

The probabilities of nucleosome occupancy were calculated along the selected sequences and averaged using the integration sites to align the sequences. As shown in Figure 7A, both MLV and HIV-1 integration sites were found locally associated with high nucleosome occupancy probability (respectively 0.73 and 0.67) showing a preference of both viruses for genomic sites occupied by a nucleosome. Comparison between nucleosome occupancy found at MLV and HIV-1 integration sites and the global occupancy mean calculated for the entire genome, or the occupancy found at randomly generated insertion sites, showed a preference of MLV for more dense region whereas HIV-1 integration is favored in regions of lower occupancy (Figure 7B). A deeper analyses of the chromatin density directly surrounding the integration site showed a global increase in the average *in vivo* nucleosome occupancy within the −5000 to +5000 bp range surrounding the MLV integration sites while a global decrease of the average nucleosome occupancy was observed around the HIV-1 insertion loci (Figure 7C). Moreover, comparison of the mean occupancy found at distal sites (1 kbp from the integration site) for MLV and HIV-1 also showed that MLV integration was preferred in region of higher nucleosome density than HIV-1 (0.739 for MLV and 0.679 for HIV-1, see Figure 7D).

Consequently, our results indicate a preferential integration into a nucleosome in infected cells for both MLV and HIV-1. However the nucleosome targeted by both viruses appear located in differently dense chromatin contexts. This shows that MLV and HIV-1 present distinct and opposite preferences for chromatin density surrounding the nucleosomal integration site supporting a modulation of retroviral integration by the chromatin density surrounding the targeted nucleosome as suggested by our *in vitro* data.

## Discussion and conclusion

Retroviral integration selectivity is thought to be regulated by viral and cellular determinants and some of them are specific of the retrovirus. Among all retroviruses, the IN protein and component of the Gag polyprotein have been shown to be involved in integration selectivity [60]. Interactions between these viral components and chromatin structure are thought to affect the selectivity of retroviral integrases. Early experiments performed *in vitro* using recombinant enzymes and reconstituted nucleosomal DNA indicated that nucleosomes were a preferential target for *in vitro* integration [42-46]. Compaction of a polynucleosome template by histone H1 was also shown to affect differently integration catalysed by two different INs (HIV and ASV) [52]. Concerted integration assays were more recently employed to investigate the influence of chromatin structure on integration site selectivity [61,36]. These data confirmed that HIV-1 global integration was favoured into nucleosomal DNA [62] and demonstrated that the chromatin structure and dynamics could influence the physiological full-site integration reaction [36]. This was then confirmed in a selectivity assay using a mixture of vectors with different nucleosome densities indicating that full-site HIV-1 integration was favoured in less dense polynucleosomal template [57]. Taken together these data obtained in different nucleosomal models with different chromatin structures and various retroviral integrases, in addition to the differences found in *in vivo* integration selectivity from one retrovirus to another one suggest that intrinsic properties of the viral intasome as well as the structure, density and dynamics of the chromatin around the targeted nucleosome play an important role in the integration site selection.

To determine the importance of this regulation in the local association of the intasome with nucleosomes, we focused our present work on the intrinsic properties of different retroviral integrases that can affect the final intasome•nucleosomal DNA functional interaction. We have chosen various retroviral integrases belonging to distinct genera displaying different *in vivo* selectivity and target sequence duplications marks. These enzymes were tested *in vitro* for their integration activity on chromatinized template in the lack of cellular cofactors. For this purpose we have selected reaction conditions allowing efficient concerted integration for all the enzymes tested in the work without the need of additional cofactors. Two main *in vitro* integration conditions were described in the literature using either long donor substrates, PEG and low enzyme concentration [48,49] either short donor substrate without PEG but requiring higher enzyme concentration and cellular cofactors, as LEDGF/p75, for efficient integration activity [50]. Since our main aim was to study the impact of chromatin on retroviral INs without their cofactors we have chosen conditions including PEG and allowing efficient integration for all the enzymes tested. To avoid biases due to the choice of these specific reaction conditions (presence of non-physiological components as PEG) we focused our analyses on the physiological full site integration products catalyzed by the retroviral enzyme. Indeed, these events are undoubtedly catalyzed only by functional intasomes and, thus, the experimental conditions were not expected to impact the choice of the integration sites. Under these conditions, we found that HIV-1, PFV, ASV and MLV enzymes were not affected equally by nucleosomes. PFV and MLV integration efficiencies were increased in a chromatinized template, whereas HIV-1 and ASV integrations were inhibited by stable and compact chromatin. Furthermore PFV and MLV integration events occurred preferentially in the 5S-G5E4 positioning region of our plasmid with a significant preference for the stably associated nucleosomes. In contrast, HIV-1 and ASV integration events were found preferred in regions of low nucleosomes occupancy especially in the less dense region of our acceptor DNA. These differences were not found dependent on the purification procedure nor reaction conditions since same profiles were always found for the various retroviral INs when purified or tested in distinct conditions (concentration, presence of detergent, use of donor DNA containing various viral U3/U5 ends combination, see Additional file 4: Figure S4). Even if we can not completely rule out any bias link to the reaction conditions selected for the study (presence of PEG for example) the focus of the analysis on the full site integration products is expected to limit this bias. Indeed, even if the formation of a catalytically proficient intasome remains a limiting step with regard to integration efficacy, the reaction conditions should only affect the amount of functional intasomes formed and not the choice of the integration site dictated both the architecture of the intasome and the local target DNA structure. This is supported by the fact that the differences in the sensitivity toward nucleosomal density were found independent on the efficiency of concerted integration. Indeed, ASV was found more active than PFV on naked DNA but was also found inhibited by stable chromatin as HIV-1 (less active than PFV in catalyzing concerted integration events).

Furthermore, the differences found between HIV-1/ASV and PFV/MLV INs were not dependent on the presence of the additional NED domain in PFV, indicating that the differential effect of chromatin on these enzymes *in vitro* is probably mainly due to local differences in the architecture of the catalytic pocket within the functional intasomes and not to global structural differences between the complexes. This is supported by the differences found between the HIV-1 integration reactions, leading to different staggered cuts in the target DNA. Indeed we previously showed that full site and half site integration could be impacted differently by nucleosome assembly *in vitro* [36]. Interestingly, differences were also found in this work regarding the effect of nucleosomes on the selectivity of HIV-1 integration reactions leading to 4 bp, 5 bp or 6 bp target DNA duplications (see Additional file 6: Figure S6). Indeed, while 5 bp and 6 bp integration reactions were highly disfavored in the stable chromatin region of the acceptor plasmid, 4 bp events were more widespread in the backbone with a clear preference for the high nucleosome density region. Since the ratio of chromatin assembly did not influence the proportion of these “non-physiological” integration events (Additional file 3: Figure S3), the latter are most likely catalyzed by aberrant intasomes structures as previously demonstrated [14]. Consequently, the most reasonable hypothesis that could account for their enrichment in nucleosome dense regions would be that the IN sensitivity toward chromatin is mainly driven by the structure of the IN/viral DNA complex that can or cannot accommodate nucleosomal DNA depending on the relative position of the active sites.

Because no bias in the PFV integration site positions was found in the naked version of the acceptor plasmid (Figure 5 and Additional file 5: Figure S5), our data indicate that the PFV and MLV intasomes can fit with compacted chromatin, in contrast to HIV-1 and ASV INs. The structure of the target DNA, and especially its bending, in the retroviral intasome is expected to be governed by the space between the two catalytic sites involved in the staggered cut leading to different target DNA duplication size [8-10]. This target DNA curvature varying in the different intasomes could, thus, impact the nucleosome sensitivity of the retroviral enzymes. This is supported by the differences found between the retroviral enzymes tested here catalyzing different target sequence duplication and by the differential effect of nucleosomes on the selectivity of HIV-1 integration reactions leading to 4 bp, 5 bp or 6 bp target DNA duplication. Interestingly, recent work highlighted the importance of the presence of flexible pyrimidine/purine steps between the attacked phosphate groups during the strand transfer reaction in promoting DNA bending and thus allowing the target DNA to adopt a conformation compatible with its capture by the intasome, with a minimal energetic expense [38] and reviewed in [39]. The extent to which the target DNA must be bent depends on the relative position of the IN active sites responsible for the pair-wise strand transfer reaction within the intasome. In the context of a nucleosome, the target DNA is already structurally constrained, therefore, its engagement into the target DNA capture complex would depend on the conformational rearrangements required, hence on the intasome structure. Indeed, this bending of the target DNA in linked to the position of the IN active sites for the pair-wise strand transfer reaction. This bendability is also expected to be affected by the nucleosome structure and chromatin environment which can allow DNA extrusion and facilitate its optimal curvature for integration. Both intrinsic structural properties of the enzymes and nucleosome structure may, thus, play an important role in the final integration site selection. Especially all these data confirm that intasome structural constraints, as the distance between catalytic sites, participate in the local selection of the integration site within chromatin by modulating the functional affinity of the integration complex with nucleosomal DNA in specific chromatin structure.

Additional interactions between intasomes and nucleosomes could also be modulated by the chromatin density at the insertion locus. Especially histone tails could be more or less accessible depending on the compaction of the surrounding nucleosomes. Comparison of the relationship between integration sites from two retroviral paradigms (HIV- and MLV) and nucleosome occupancy in cells (Figure 7) indicates that, both retroviral insertion loci were associated locally with high nucleosome occupancy probability. This suggests that in both cases the selected integration site is probably occupied by a nucleosome which is in contrast to what was observed for HIV-1 in nucleosome dense regions of the receptor templates used *in vitro*. The apparent discrepancy found for the local preference of HIV-1 IN for nucleosomes between *in vitro* and *in vivo* data could be due to the fact that, in the cells, additional remodeling machineries could be involved during the integration process, especially in the highly transcribed regions of viral DNA insertion as supported by previous data [36], as well as the lack of cellular tethering cofactors in the biochemical assays used in this work. However, when the chromatin structure surrounding the integration sites was analyzed a significant preference for region of higher nucleosome occupancy was found for MLV comparing to HIV-1 IN that preferred less dense regions of the host DNA. This cellular phenotype appears compatible with the intrinsic sensitivity of the retroviral enzymes toward nucleosomes and their capability to accommodate dense or less dense chromatin structure as found *in vitro*. Thus, even in cellular context, when targeting factors are present, the IN-specific preference found *in vitro* for regions of variable nucleosomes density can also be detected. Even if the role of the IN cofactors in the local association between intasome and nucleosome remains to be established, our data suggests that the intrinsic sensitivity of retroviral INs toward specific chromatin structure may play an important role in the local selection of the integration site in infected cells in addition to their targeting toward specific region of the host genome by the host cofactors. Taken together all these data support a preferential retroviral integration into a nucleosome but in different chromatin contexts in a mechanism that will depend both on the integrases structures and modulations by the chromatin environment of the targeted nucleosome.

Consequently, the retroviral integration targeting toward nucleosomes appears as a multiple steps process that includes a targeting of the retroviral intasome toward suitable regions of the chromatin as promoter or gene body due to specific interaction between INs and their targeting factors as LEDGF/P75 or BET proteins; and a more local targeting toward the suitable integration site governed by the intrinsic sensitivity of the retroviral INs to specific target DNA bending and chromatin density surrounding the integration locus. These last steps are expected to be governed by the physical constraints within the intasomes modulating its functional interaction with the targeted nucleosome. This model is strongly supported by several data from the literature. Knock out experiments have previously established that LEDGF/p75 was not essential for HIV-1 integration *in vivo* [33] and, in the absence of LEDGF/p75, the weak consensus associated with HIV-1 integration is maintained and integration is (though to a lower extent) still associated to active transcription units. Furthermore, a C-terminal deletion derivative of MLV IN, unable to bind BET proteins, still allowed integration catalysis, *in vivo*, and does not show changes in its local integration selectivity [37]. These data, in addition to our work, support the dependence on the structure of the intasome in its functional association with nucleosomes after the initial targeting step mediated by cellular cofactors (*e.g.* LEDGF/p75 and BET proteins). Consequently, taken together, these results point out the retroviral intasome, itself, as an important actor in the integration site selection process.

Other additional key parameters are also known to be important in integration site selection, such as the three dimensional localization of the chromatin in the nucleus in comparison to the PIC nuclear entry site [63] or the involvement of chromatin remodeling proteins that can be found in the integration locus area and expected to affect the nucleosomes stability. Global analyses of the HIV-1 integration site showed that integration occurs in the active region of the chromatin carrying nucleosomes bearing the H3K36me3 modification that promotes transcription and remodeling [55]. *In silico* comparison of the integration sites found in infected cells and nucleosome positioning show that the process occurs in a region of low nucleosomes density within the chromatin [36]. These data suggest that, for HIV-1 integration to occur, a nucleosomal DNA structure could be required at the integration site but in a context of dynamic chromatin. In this context, the DNA extrusion from the nucleosome is expected to be facilitated, especially during remodeling, allowing the required degree of bending. The position of the integration sites on the surface of the nucleosome [55] also suggests that other proteins inducing local remodeling of the DNA in the nucleosome nucleoprotein complex and/or supplementary intasome/nucleosome interactions could be involved, in addition to the anchoring factor LEDGF/p75. This would makes the cellular integration process more complex than just involving a direct binding of the retroviral intasomes to the DNA at the nucleosome surface and would highlight the importance of the chromatin context around the targeted nucleosome modulating additional contacts between the integration partners.

## Methods

### Proteins

PFV and ASV INs were purified from *E.coli* BL21 bacteria using the same procedure than described previously [47] [64]. HIV-1 IN was purified either from bacteria using the same procedure as described for PFV enzyme [47] either from yeast was purified as previously reported [65]. MLV IN was purified using the procedure reported by [66].

### DNA substrates

Both pBSK-5SG5E4 (p5S) target and HIV-1 donor DNA were described previously [36]. The ASV and PFV 250 bp donor DNA were obtained by amplifying the SupF-containing fragment from the pUC19-SupF vector described before [14] using primers containing either the 20 bp from the ASV U3 viral ends or PFV U5 viral ends or U3 and U5 MLV ends:

- ASV_U3_SupF5′ 5′AATGTAGTCTTATGCAATACTCTTGTAGTCTTGCAATTAACGTTGCCCGGATCCGGTCGCGC 3′, ASV_U3_SupF3′ 5′AATGTAGTCTTATGCAATACTCTTGTAGTCTTGCAAGCGGCGCGTCATTTGATATGATGCG 3′PFVSupF5′ 5′ ATTGTCATGGAATTTTGTATATTGATTATCCTTTAACGTTGCCCGGATCCGGTCGCGC 3′, PFVSupF3′ 5′ ATTGTCATGGAATTTTGTATATTGATTATCCTGCGGCGCGTCATTTGATATGATGCG 3′. MLVSupF5′ 5′TATGAAAGACCCCACCTGTAGTTAACGTTGCCCGGATCCGGTCGCGC 3′
- MLVSupF3′ 5′ TATGAAAGACCCCCGCTGACGCGGCGCGTCATTTGATATGATGCG 3′, respectively.

The amplified fragments were purified from a 1% agarose gel using the PROMEGA Wizard SV gel and PCR cleanup system and 5′ radiolabelled using the T4 polynucleotide DNA kinase (PROMEGA). The different donor DNA are described in Additional file 1: Figure S1.

Polynucleosome templates were assembled using purified HeLa core histones (H3, H4, H2A and H2B) [67] by gradient salt dialysis [68]. Structure of regions I and II of the p5S vector was previously checked by *ab initio* prediction of nucleosome occupancy throughout the DNA sequence performed by computing the free-energy landscape associated with the bending of DNA around histone octamers to form nucleosomes [36] (see Figure 1). Nucleosome assembly was checked by DNase I protection, REA assay and mono- and di-nucleosome gel shift, as done before [36] and described in Additional file 2: Figure S2.

### Concerted integration

Standard concerted integration reactions were optimized from conditions described previously [65] in order to allow efficient catalyse of full site integration reaction in absence of other cellular cofactors. For this purpose we used 5′-end-labeled donor DNA (10 ng) and naked or chromatinized circular target DNA plasmids (50 ng) and purified INs (typically 100 nM) previously diluted to 1 μM in 1 M NaCl, 20 mM HEPES 1 M pH7, 10 mM DTT for 30 minutes on ice. Then 200 nM IN were incubated 30 minutes on ice with 10 ng of donor DNA and 50 ng of acceptor plasmid in 5 μl final volume. Reaction was then started by adding 5 μl of the reaction buffer (final concentrations 100nM IN, 15% DMSO, 8% PEG, 10 mM MgCl2, 20 μM ZnCl2, 100 mM NaCl, 10 mM DTT). After the reaction, integration products were loaded onto 1% agarose gel. The gel was then dried and autoradiographied. Quantification of the integration activity was performed using the Image J software with the following procedure: the bands corresponding to the free substrate (S), the donor/donor (d/d), linear FSI (FSI) and circular HSI + FSI (HSI + FSI) were quantified. The percentage of HSI + FSI integration activity was calculated as (HSI + FSI)/[(FSI) + (HSI + FSI) + (d/d) + (S)] × 100. Percentage of FSI integration activity was determined as (FSI)/[(FSI) + (HSI + FSI) + (d/d) + (S)] × 100. The global FSI activity was estimated from the amount of linear FSI products which has previously been shown to be representative of the circular FSI form. The circular FSI reaction efficiency was then quantified by cloning the integration products in bacteria according to the same protocol as described previously [14] with some minor modifications. The integration reaction performed with unlabelled donor DNA and the corresponding acceptor plasmids was 15 times scaled up and the integration products were loaded on 1% agarose gel after deproteinization using protease K 1 mg/ml (ROCHE) and phenol/chloroform/isoamyl alcohol (24/25/1, v/v/v). The open circular forms of the products were purified using the PROMEGA Wizard SV gel and PCR cleanup system and 2–5 μl aliquots of the purified DNA were used to transform electrocompetent MC1060/P3 *E. coli* strain which contained ampicillin-, tetracycline- and kanamycin-resistance genes. Integration clones carrying the *supF* gene were selected in the presence of 40 μg/ml ampicillin, 10 μg/ml tetracycline and 15 μg/ml kanamycin. Integration loci determination was performed by isolating plasmids from triple-resistant colonies and PCR sequencing (ABI Prism big dye terminator cycle sequencing ready reaction kit, Applied Biosystems) using the corresponding HIV-1, PFV, ASV and MLV primers described above. The number of experiments was adjusted to reach the required amount of integrants for statistically significant analyses.

### Nucleosome occupancy prediction

Nucleosome occupancy prediction was determined using the method previously described in [53] and used in [36].

## Additional files

Additional file 1: Figure S1. Structure of the viral donor substrates. All the donor DNA contain the SupF amber suppressor gene required for cloning of the integrants and carry the specific HIV-1, PFV, ASV or MLV U3 or U5 viral ends sequences. Additional file 2: Figure S2. Structure of the pBSK-zeo-5S-G5E4 (p5S) acceptor plasmid. A- Nucleosome occupancy prediction and restriction site positions determined using the method previously described by [53] and used in [36]. Restriction sites localized either in the 5S-G5E4 fragment or in the pBSK-Zeo (pZeo) backbone were plotted in the graph. The number of sites is also reported as well as their position in the vector sequence. B- Analysis of the global nucleosomal structure of the plasmid by DNase 1 protection. 200 ng of naked p5S (lanes 1) or chromatinized with 1/1.1 (lanes 2) or 1/1.3 (lanes 3) DNA histones ratios were digested using 0.15 units of DNase 1 for 2 minutes at 37°C. Samples were then submitted to deproteinization using a 24/25/1 (v/v/v) phenol/chloroform/isoamyl alcohol solution and loaded onto 1% agarose gel 1% SybrSafe. The percentage of cleaved DNA was quantified and plotted in the graph. C- Restriction enzyme assay analysis of the p5S nucleosomal structure. 200 ng of naked p5S (lanes 1) or chromatinized with 1/1.1 (lanes 2) or 1/1.3 (lanes 3) DNA histone ratios were digested with 1 unit of the corresponding restriction enzyme for 30 minutes at 37°C. The product was then loaded onto 1% agarose gel 1% SybrSafe and the restriction band was quantified using ImageJ software and plotted as percentage of DNA cut. Enzymes cleaving in the 5S-G5E4 region on a nucleosome, in the linker sequence or in the pZeo backbone were chosen. D- Agarose nucleosome gel shift assay. 200 ng of naked p5S (lanes 1) or chromatinized with 1/1.1 (lanes 2) or 1/1.3 (lanes 3) DNA histones ratios were digested by *Eco*RI and loaded onto 0.8% agarose gel under non-denaturating conditions. The position of the free nucleosome positioning fragment is plotted (free DNA) as well as the position of the mononucleosome (MN) and polynucleosome (PN) fragments. Additional file 3: Figure S3.
*In vitro* integration “fidelity” of the different retroviral integrases studied. The target site duplications found in integrants clones selected after integration assay performed in p5S naked or 1/1.3 chromatinized vector were sequenced and plotted for all integrases. The percentage of clones carrying the corresponding duplication is reported as the mean of 3 independent experiments. Additional file 4: Figure S4. A- Effect of the integrase expression and purification conditions on *in vitro* concerted integration. HIV-1 expressed in bacteria or yeast as native enzyme or Ct (His)_6_ tag fusion protein purified following the same protocol than PFV integrase were assayed on standard *in vitro* concerted integration on the p5S naked or chromatinized with 1/1.1 or 1/1.3 DNA/histones ratios. After separating of the integration products on agarose gels the different forms were quantified using ImageJ and reported as percentage of integration (100% correspond to the integration activity found on naked DNA). Results are reported as the mean ± standard deviation (error bars) of at least three independent sets of experiments. B- Effect of the viral DNA sequence in the donor DNA on *in vitro* concerted integration. Concerted integration assays were performed using HIV-1 IN and viral donor DNA containing either U3/U5, U3/U3 or U5/U5 viral ends sequence. After separating of the integration products on agarose gels the different forms were quantified using ImageJ and reported as percentage of integration (100% correspond to the integration activity found on naked DNA). Results are reported as the mean ± standard deviation (error bars) of at least three independent sets of experiments. Additional file 5: Figure S5. Distribution of PFV (A), HIV-1 (B), MLV (C) and ASV (D) integration sites on naked pBSK-zeo-G5E4 (p5S) vector. Fifty integrants carrying the correct target DNA duplication obtained after integration assay carried on the naked p5S vector localized in the 5S-G5E4 fragment were positioned the DNA sequence and compared to the nucleosome occupancy determined using the method previously described by [53] and used in [36]. Additional file 6: Figure S6. HIV-1 integration products formed *in vitro* are differently affected by the nucleosomal DNA structure. Two hundred selected clones obtained after HIV-1 integration in naked and chromatinized vectors were sequenced and the structure of the target DNA duplication found at the integration locus was shown as percentage clones carrying correct 5 bp duplications, 4, 6 bp duplications and other structures (A). The positions of the different integration events were identified and shown in region 1 or region 2 in the naked (B) or nucleosomal acceptor plasmid (C). The values correspond to the mean ± standard deviation (error bars) of 3 to 6 independent sets of experiments. A Student test was performed on serial values and the significant p values are reported in the figure.
