# Supplementary figures and images for: Intasome architecture and chromatin density modulate retroviral integration into nucleosome
Source: Retrovirology. 2015 Feb 7;12:13. doi: 10.1186/s12977-015-0145-9 (PMC4358916; doi:10.1186/s12977-015-0145-9)

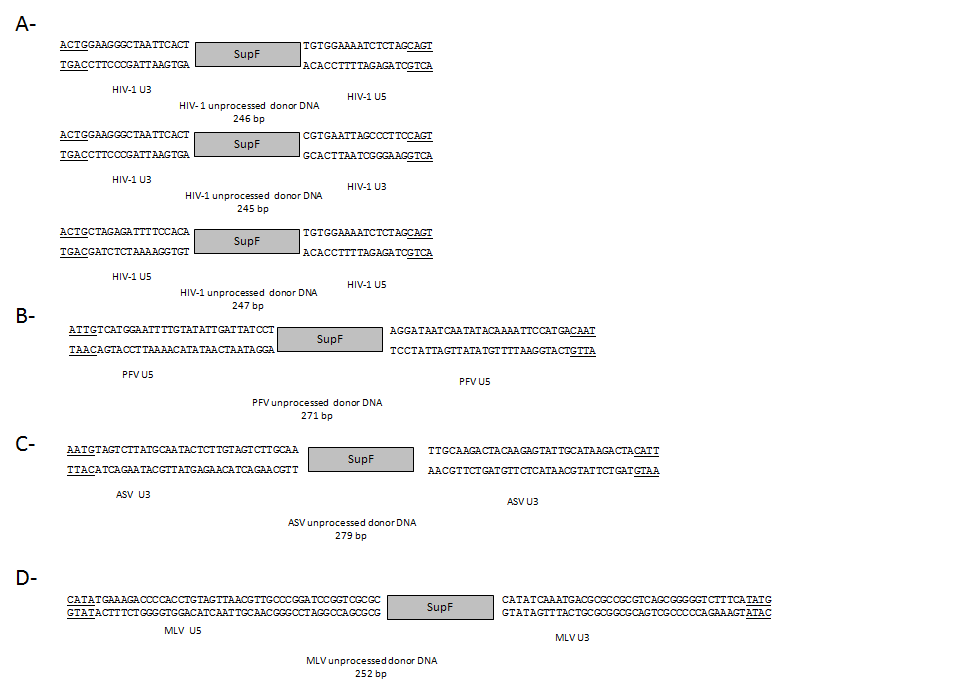

Supplement: Additional file 1: Figure S1. — Structure of the viral donor substrates. All the donor DNA contain the SupF amber suppressor gene required for cloning of the integrants and carry the specific HIV-1, PFV, ASV or MLV U3 or U5 viral ends sequences. [file 12977_2015_145_MOESM1_ESM.tiff]

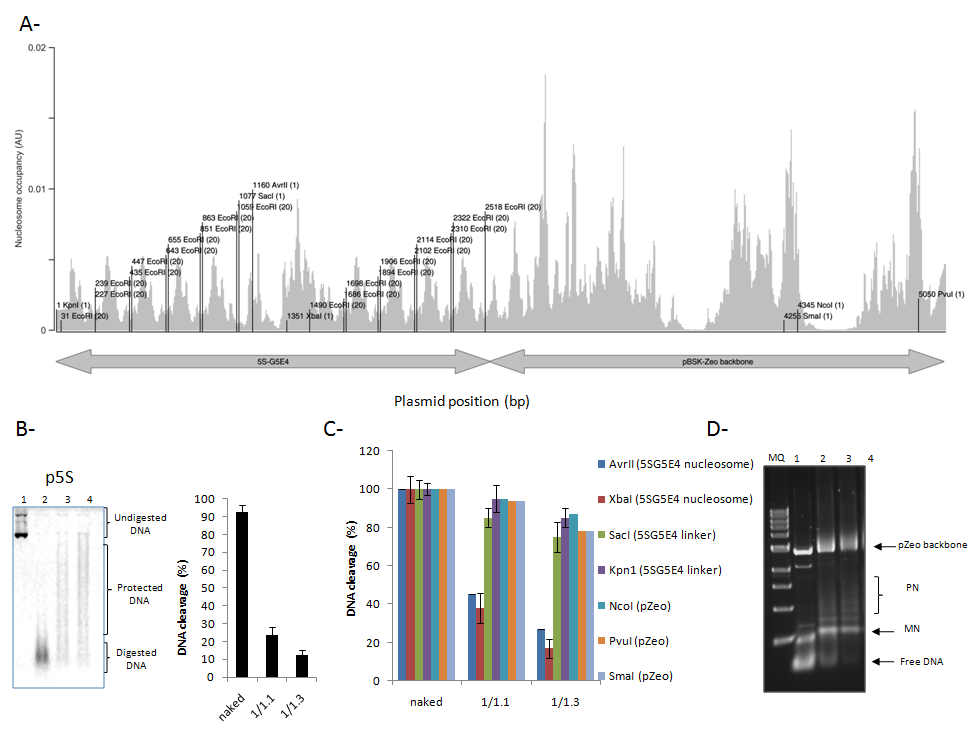

Supplement: Additional file 2: Figure S2. — Structure of the pBSK-zeo-5S-G5E4 (p5S) acceptor plasmid. A- Nucleosome occupancy prediction and restriction site positions determined using the method previously described by [53] and used in [36]. Restriction sites localized either in the 5S-G5E4 fragment or in the pBSK-Zeo (pZeo) backbone were plotted in the graph. The number of sites is also reported as well as their position in the vector sequence. B- Analysis of the global nucleosomal structure of the plasmid by DNase 1 protection. 200 ng of naked p5S (lanes 1) or chromatinized with 1/1.1 (lanes 2) or 1/1.3 (lanes 3) DNA histones ratios were digested using 0.15 units of DNase 1 for 2 minutes at 37°C. Samples were then submitted to deproteinization using a 24/25/1 (v/v/v) phenol/chloroform/isoamyl alcohol solution and loaded onto 1% agarose gel 1% SybrSafe. The percentage of cleaved DNA was quantified and plotted in the graph. C- Restriction enzyme assay analysis of the p5S nucleosomal structure. 200 ng of naked p5S (lanes 1) or chromatinized with 1/1.1 (lanes 2) or 1/1.3 (lanes 3) DNA histone ratios were digested with 1 unit of the corresponding restriction enzyme for 30 minutes at 37°C. The product was then loaded onto 1% agarose gel 1% SybrSafe and the restriction band was quantified using ImageJ software and plotted as percentage of DNA cut. Enzymes cleaving in the 5S-G5E4 region on a nucleosome, in the linker sequence or in the pZeo backbone were chosen. D- Agarose nucleosome gel shift assay. 200 ng of naked p5S (lanes 1) or chromatinized with 1/1.1 (lanes 2) or 1/1.3 (lanes 3) DNA histones ratios were digested by EcoRI and loaded onto 0.8% agarose gel under non-denaturating conditions. The position of the free nucleosome positioning fragment is plotted (free DNA) as well as the position of the mononucleosome (MN) and polynucleosome (PN) fragments. [file 12977_2015_145_MOESM2_ESM.tiff]

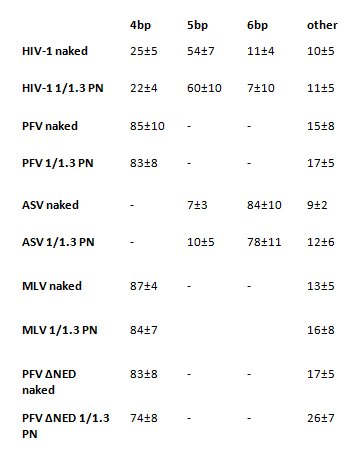

Supplement: Additional file 3: Figure S3. — In vitro integration “fidelity” of the different retroviral integrases studied. The target site duplications found in integrants clones selected after integration assay performed in p5S naked or 1/1.3 chromatinized vector were sequenced and plotted for all integrases. The percentage of clones carrying the corresponding duplication is reported as the mean of 3 independent experiments. [file 12977_2015_145_MOESM3_ESM.tiff]

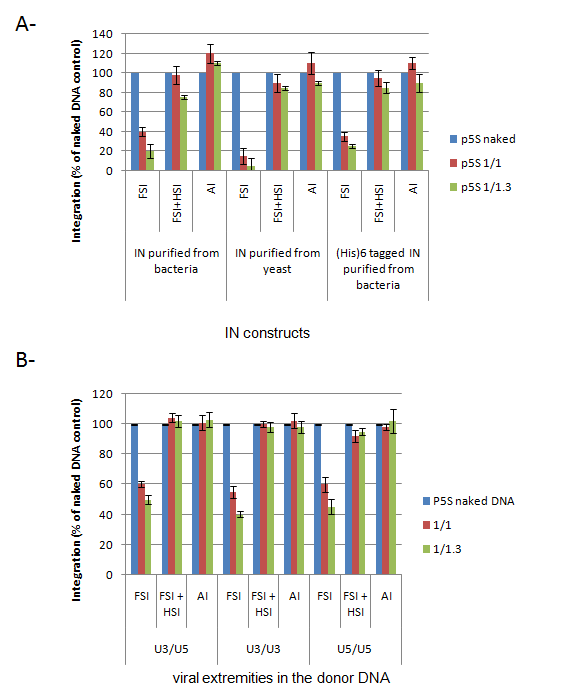

Supplement: Additional file 4: Figure S4. — A- Effect of the integrase expression and purification conditions on in vitro concerted integration. HIV-1 expressed in bacteria or yeast as native enzyme or Ct (His)6 tag fusion protein purified following the same protocol than PFV integrase were assayed on standard in vitro concerted integration on the p5S naked or chromatinized with 1/1.1 or 1/1.3 DNA/histones ratios. After separating of the integration products on agarose gels the different forms were quantified using ImageJ and reported as percentage of integration (100% correspond to the integration activity found on naked DNA). Results are reported as the mean ± standard deviation (error bars) of at least three independent sets of experiments. B- Effect of the viral DNA sequence in the donor DNA on in vitro concerted integration. Concerted integration assays were performed using HIV-1 IN and viral donor DNA containing either U3/U5, U3/U3 or U5/U5 viral ends sequence. After separating of the integration products on agarose gels the different forms were quantified using ImageJ and reported as percentage of integration (100% correspond to the integration activity found on naked DNA). Results are reported as the mean ± standard deviation (error bars) of at least three independent sets of experiments. [file 12977_2015_145_MOESM4_ESM.tiff]

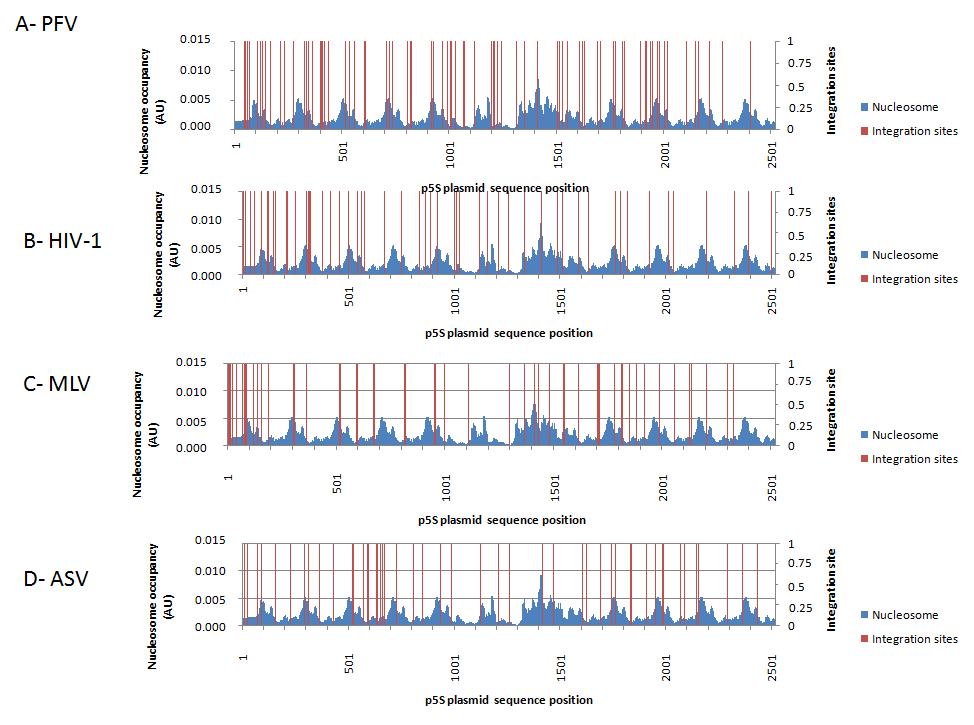

Supplement: Additional file 5: Figure S5. — Distribution of PFV (A), HIV-1 (B), MLV (C) and ASV (D) integration sites on naked pBSK-zeo-G5E4 (p5S) vector. Fifty integrants carrying the correct target DNA duplication obtained after integration assay carried on the naked p5S vector localized in the 5S-G5E4 fragment were positioned the DNA sequence and compared to the nucleosome occupancy determined using the method previously described by [53] and used in [36]. [file 12977_2015_145_MOESM5_ESM.tiff]

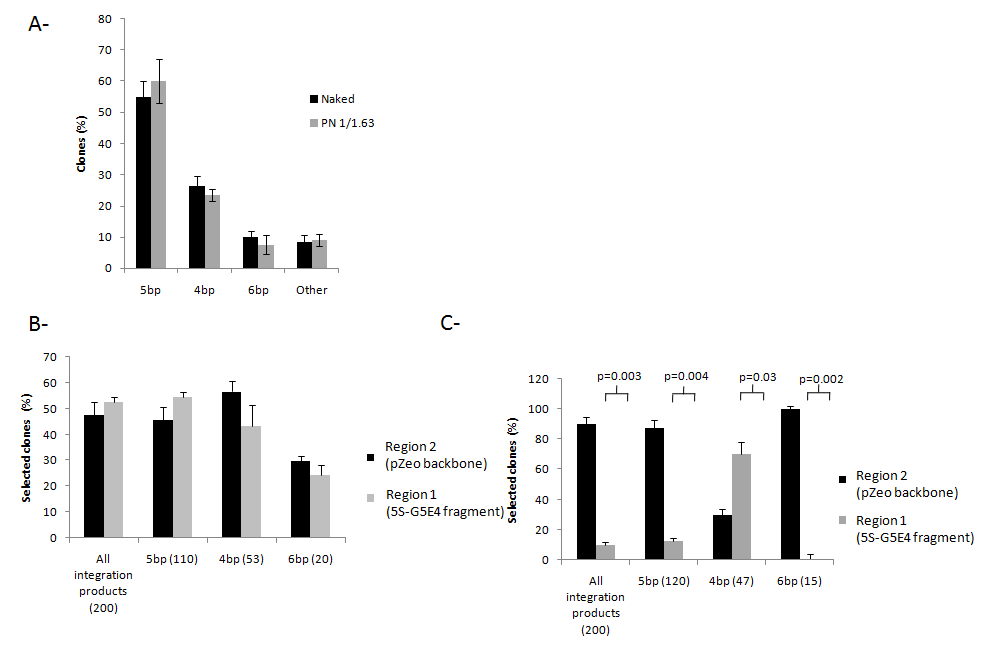

Supplement: Additional file 6: Figure S6. — HIV-1 integration products formed in vitro are differently affected by the nucleosomal DNA structure. Two hundred selected clones obtained after HIV-1 integration in naked and chromatinized vectors were sequenced and the structure of the target DNA duplication found at the integration locus was shown as percentage clones carrying correct 5 bp duplications, 4, 6 bp duplications and other structures (A). The positions of the different integration events were identified and shown in region 1 or region 2 in the naked (B) or nucleosomal acceptor plasmid (C). The values correspond to the mean ± standard deviation (error bars) of 3 to 6 independent sets of experiments. A Student test was performed on serial values and the significant p values are reported in the figure. [file 12977_2015_145_MOESM6_ESM.tiff]
